# Intrinsic factors and the embryonic environment influence the formation of extragonadal teratomas during gestation

**DOI:** 10.1186/s12861-015-0084-7

**Published:** 2015-10-09

**Authors:** Constantinos Economou, Anestis Tsakiridis, Filip J. Wymeersch, Sabrina Gordon-Keylock, Robert E Dewhurst, Dawn Fisher, Alexander Medvinsky, Andrew JH Smith, Valerie Wilson

**Affiliations:** MRC Centre for Regenerative Medicine, School of Biological Sciences, SCRM Building, The University of Edinburgh, Edinburgh bioQuarter, 5 Little France Drive, Edinburgh, EH16 4UU UK; Drug Discovery Unit, Telethon Kids Institute, PO Box 855, West Perth, WA 6872 Australia

**Keywords:** Extragonadal teratoma, Pluripotency, Oct4, Nanog, Brachyury, Inducible expression

## Abstract

**Background:**

Pluripotent cells are present in early embryos until the levels of the pluripotency regulator Oct4 drop at the beginning of somitogenesis. Elevating Oct4 levels in explanted post-pluripotent cells in vitro restores their pluripotency. Cultured pluripotent cells can participate in normal development when introduced into host embryos up to the end of gastrulation. In contrast, pluripotent cells efficiently seed malignant teratocarcinomas in adult animals. In humans, extragonadal teratomas and teratocarcinomas are most frequently found in the sacrococcygeal region of neonates, suggesting that these tumours originate from cells in the posterior of the embryo that either reactivate or fail to switch off their pluripotent status. However, experimental models for the persistence or reactivation of pluripotency during embryonic development are lacking.

**Methods:**

We manually injected embryonic stem cells into conceptuses at E9.5 to test whether the presence of pluripotent cells at this stage correlates with teratocarcinoma formation. We then examined the effects of reactivating embryonic Oct4 expression ubiquitously or in combination with Nanog within the primitive streak (PS)/tail bud (TB) using a transgenic mouse line and embryo chimeras carrying a PS/TB-specific heterologous gene expression cassette respectively.

**Results:**

Here, we show that pluripotent cells seed teratomas in post-gastrulation embryos. However, at these stages, induced ubiquitous expression of Oct4 does not lead to restoration of pluripotency (indicated by Nanog expression) and tumour formation *in utero*, but instead causes a severe phenotype in the extending anteroposterior axis. Use of a more restricted T(Bra) promoter transgenic system enabling inducible ectopic expression of Oct4 and Nanog specifically in the posteriorly-located primitive streak (PS) and tail bud (TB) led to similar axial malformations to those induced by Oct4 alone. These cells underwent induction of pluripotency marker expression in Epiblast Stem Cell (EpiSC) explants derived from somitogenesis-stage embryos, but no teratocarcinoma formation was observed *in vivo*.

**Conclusions:**

Our findings show that although pluripotent cells with teratocarcinogenic potential can be produced in vitro by the overexpression of pluripotency regulators in explanted somitogenesis-stage somatic cells, the *in vivo* induction of these genes does not yield tumours. This suggests a restrictive regulatory role of the embryonic microenvironment in the induction of pluripotency.

**Electronic supplementary material:**

The online version of this article (doi:10.1186/s12861-015-0084-7) contains supplementary material, which is available to authorized users.

## Background

In mammals, pluripotency, the ability to generate derivatives of all three embryonic germ layers, is an essential characteristic of cells in the preimplantation and very early postimplantation epiblast as well as their in vitro counterparts, embryonic stem (ES) and Epiblast Stem (EpiSC) cells respectively. The maintenance of a pluripotent state is mediated by the activity of a core transcriptional network dominated by the principal pluripotency factors Oct 4, Nanog and Sox2 (reviewed in [1]. Shortly after the start of gastrulation, although cell fate becomes regionalised [2], epiblast cells remain pluripotent until the beginning of somitogenesis [3]. We have previously shown that ectopic Oct4 expression is the minimal requirement for reinstating pluripotency in normally non-pluripotent somitogenesis-stage embryonic cells explanted in vitro in EpiSC conditions [3]. Furthermore, simultaneous ectopic expression of only four pluripotency factors is sufficient to reprogram embryonic fibroblasts or adult somatic cells to an ES cell-like pluripotent state [4, 5].

A key feature of all pluripotent cells is their capacity to seed teratocarcinomas after engraftment into permissive sites in the adult such as the kidney capsule. Teratocarcinomas comprise differentiated tissues derived from all three germ layers as well as a self-renewing, pluripotent component, embryonal carcinoma (EC) cells [6]. In humans, teratocarcinomas and teratomas, their more benign versions lacking obvious EC cells, are classified as germ cell tumours as, by puberty, they mainly occur in the testis and ovary (for a review see [7]). In neonates, however, the majority of teratomas are extragonadal and occur along the midline, most frequently in the sacrococcygeal region [8, 9]. This may indicate that primordial germ cells (PGCs) initiate these tumours, as they pass along the midline to reach the genital ridges. An alternative hypothesis is that a somatic cell type may acquire pluripotency resulting in teratocarcinogenesis. The midline location of these tumours might in this case indicate either a susceptible somatic cell type or a permissive environment [10, 11]. In support of the latter hypothesis, ectopic Oct4 reactivation in somatic cells from somitogenesis-stage embryos grafted to the adult kidney capsule has been shown to induce teratocarcinoma formation [3]. Furthermore, the transient induction of the main reprogramming factors, Oct4, Sox2, Klf4 and c-Myc *in vivo* drives the emergence of teratomas in a variety of somatic cell types in the adult mouse [12, 13]. Nevertheless, injection of pluripotent cells into a compatible embryonic environment, the blastocyst [14] or postimplantation embryo [15] leads to assimilation of those cells into normal development. Thus the genesis of teratomas/teratocarcinomas depends on the activity of a minimal set of pluripotency factors combined with a permissive environment.

The predominant occurrence of extragonadal teratomas/teratocarcinomas in human neonates suggests that pluripotent cells are tumour-forming even during gestation. Here we sought to define whether the mouse embryo is permissive for teratocarcinoma formation on ectopic expression of pluripotency factors. We found that although injected ES cells efficiently induce tumour formation in midgestation embryos, ubiquitous or axial progenitor-specific ectopic expression of pluripotency factors in somitogenesis-stage embryos did not lead to the generation of tumours. Instead, severe developmental abnormalities occurred along the developing axis. Our findings suggest that while the somitogenesis-stage embryonic environment can suppress the neoplastic potential of cells ectopically expressing individual pluripotency factors, once pluripotency is established, pluripotent cells are capable of seeding extragonadal teratomas during gestation.

## Results

### Induction of teratomas by pluripotent cells

**Fig. 1:**
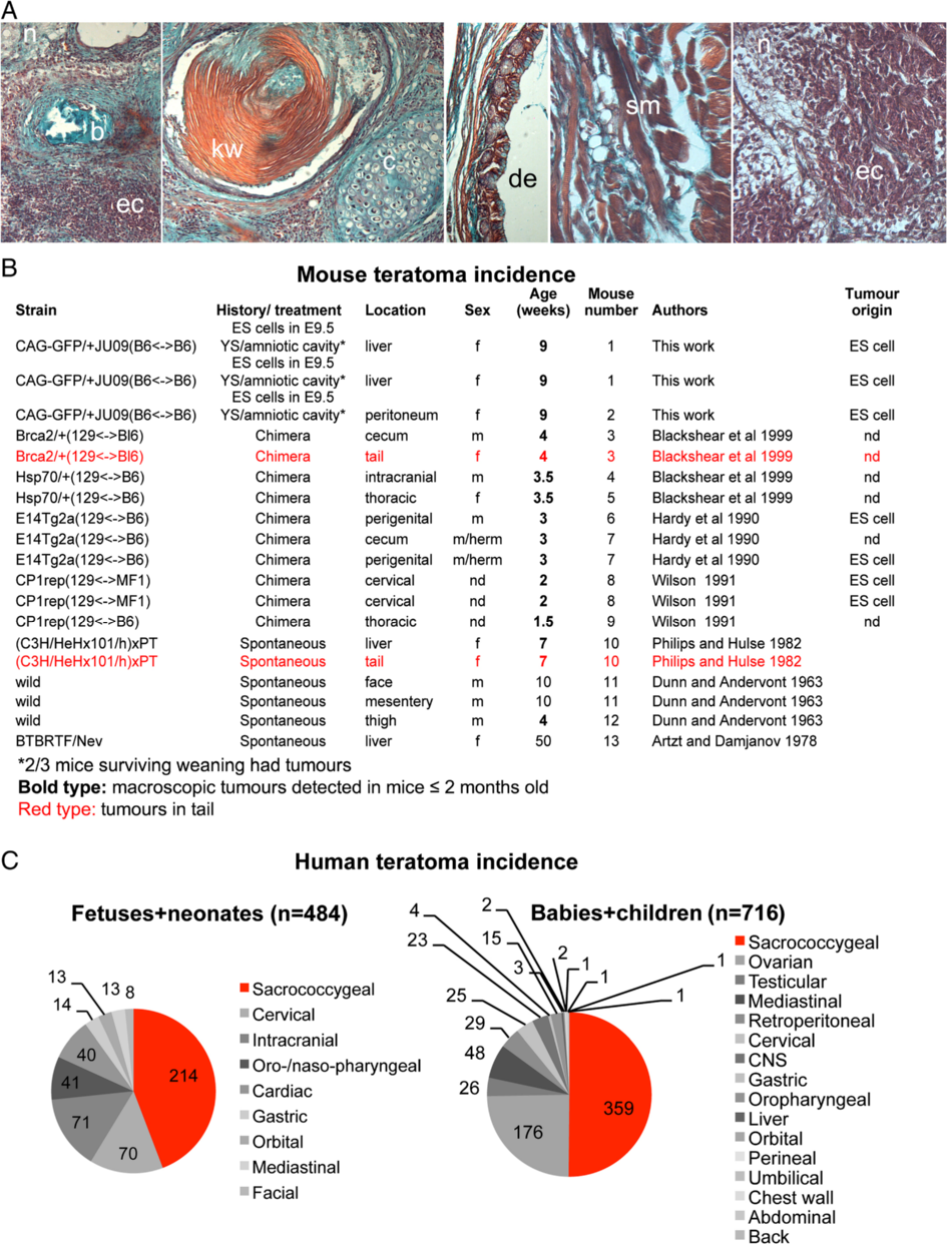
Pluripotent cells and the location of extragonadal teratomas in mice and humans. **a** Teratomas formed after manual injection of ES cells to the yolk sac and/or amniotic cavity of E9.5 embryos. ec, embryonal carcinoma; n, neural tissue; b, bone; kw, keratin whorl; c, cartilage; de, digestive epithelium; sm, striated (skeletal) muscle. **b** Locations and ages of mice developing teratomas, in this work and reported in the literature. Individual animals are assigned an arbitrary identifier (‘mouse number’) to account for the presence of multiple tumours in some animals. f, female; m, male; herm, hermaphrodite; nd, not determined. **c** Summary of teratoma location in humans (fetal and neonatal data taken from [57]; perinatal and childhood data from [35]). Data in piecharts is arranged clockwise starting with most frequent location at 12 o’clock

Manual injection of ES cell suspensions into conceptuses (either the yolk sac or amniotic cavity) at E9.5 led to the appearance after birth of extragonadal teratocarcinomas in 2/3 injected pups (Fig. 1a, b) (7 embryos were injected in total of which 5 were born and 3 pups survived to weaning), showing that ectopic pluripotent cells can seed teratomas during embryogenesis. No tumours were observed when non-pluripotent primary somatic cells from E8.5-14.5 embryos were injected into age-matched embryos embryos (BL6 or other) (approx. 200 embryos were assessed after birth, data not shown). Furthermore, a survey of the literature showed that, while reports of spontaneously occurring extragonadal teratomas are extremely rare, they can occur upon injection of ES cells to blastocysts (9/13 reported mice). In one case, they arose from a cell line known to harbour a large chromosome 8 deletion [16], a karyotypic abnormality frequently encountered in ES cell lines [17]. Interestingly, such tumours were generally found in young mice under 9 weeks old (11/13 mice; of which 8 were 1.5-4 week old pups/weanlings). Tumours were detected in multiple sites in the body (Fig. 1b), including two instances of tumours in the tail. In humans, extragonadal teratomas are often apparent at birth or in the early neonatal period (Fig. 1c), and while they are also found at multiple sites, some of which overlap with those in mice, the predominant location is at the base of the spine, in the sacrococcygeal region. Collectively, these data indicate that persistence of pluripotent cells after the natural shutdown of embryonic pluripotency is likely to result in the formation of extragonadal teratocarcinomas/teratomas shortly after birth and suggest that the posterior midline (the primitive streak and tail bud) represents a permissive environment for the manifestation of a pluripotent phenotype.

### Coincidence of pluripotency transcription factors in the posterior of somitogenesis-stage embryos

**Fig. 2:**
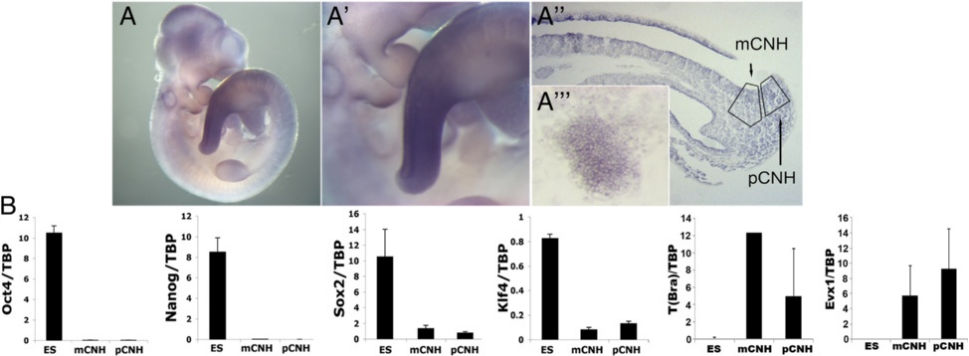
Pluripotency factor expression in the somitogenesis stage embryo. **a** Klf4 mRNA expression in an E10.5 mouse embryo analysed by wholemount *in situ* hybridisation. (*a’*) Magnified detail from image *a’* showing the increased presence of Klf4 transcripts at the posterior. (*a”*) Embryo section showing Klf4 expression within the chordoneural hinge (mCNH) of the E10.5 tailbud. pCNH, region immediately posterior to CNH. (*a”’*) Inset: Klf4 *in situ* hybridisation in undifferentiated ES cells (**b**) qPCR analysis of the expression of the main pluripotency factors and positive control genes (*T(Bra)* and *Evx1*) in CNH regions microdissected from an E10.5 (32–35 somite) embryo (their locations indicated  in *a”*). Expression levels are represented as relative to TBP. Error bars represent s.d. Mouse ES cells were included as a positive control

We hypothesised that if the acquisition or persistence of pluripotency in somatic cells in post-gastrulation stage embryos correlates with teratocarcinoma formation in extragonadal sites then it is likely to occur in locations exhibiting pluripotency factor expression. We thus examined the presence of the main pluripotency/reprogramming factors in somitogenesis stage embryos. We have shown that *Nanog* expression disappears around the end of gastrulation while *Oct4* becomes absent from somatic tissues by the ~10-somite (s) stage [3] although, like *Nanog*, its expression is retained in the PGCs [18, 19]. Expression of *Klf4*, one of the original factors necessary for reprogramming to induced pluripotency, was undetectable during early somitogenesis (data not shown). However, *in situ* hybridisation analysis revealed that *Klf4* transcripts were present at later somite stages (32-35 s) predominantly in the posterior end of the embryo up to the TB (Fig. 2a). Furthermore, we found by real-time qPCR that the chordoneural hinge (CNH), a site within the TB shown to harbour progenitor cells with neuromesodermal (NM) potency [20], also exhibited *Klf4* expression (Fig. 2b). The same region was also found by qPCR to be positive for *Sox2* expression while being devoid of *Oct4* and *Nanog* transcripts (Fig. 2b). These data combined with the reported presence of the other main reprogramming factor *c-Myc* in the posterior end of the embryo [21] indicate that there is a coincidence of pluripotency/reprogramming factors in this location, although two major pluripotency transcription factors, *Oct4* and *Nanog*, are absent.

### Ubiquitous induction of Oct4 expression *in vivo* causes severe developmental abnormalities but does not reactivate the pluripotency indicator Nanog

**Fig. 3:**
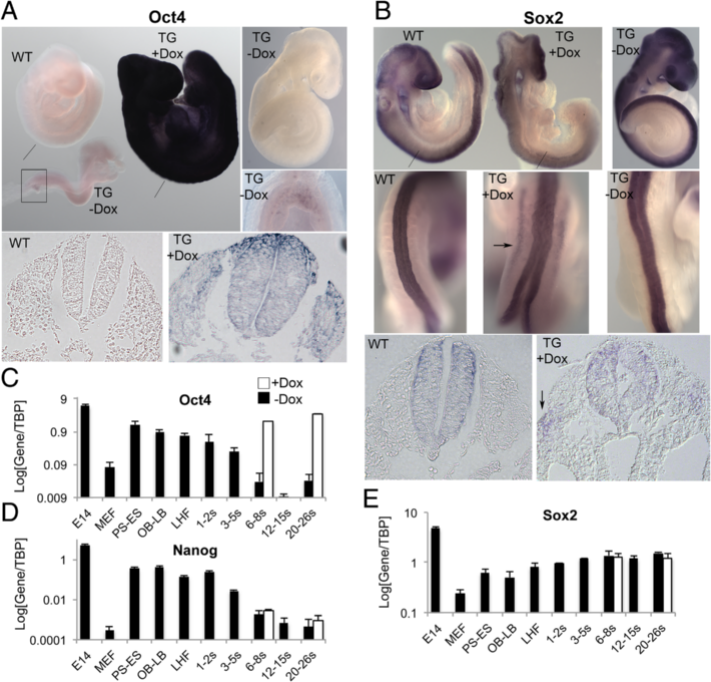
Induction of ubiquitous ectopic Oct4 expression. **a** *Top*: *Oct4* mRNA expression in wild type (wt) and induced (+Dox) or un-induced (−Dox) *TgOct4* transgenic (TG) somitogenesis embryos analysed by whole mount *in situ* hybridisation. A high magnification image of *Oct4* expression in the PGCs of the transgenic, un-induced E8.5 embryo is also shown on the right corresponding to the boxed area. Bottom: representative sections showing *Oct4* expression in the wild type (*left*) and the induced transgenic (*right*) embryos. The position of the sections relative to the embryo is indicated by the lines on the top left image. **b** *Top*: *Sox2* mRNA expression in wild type (wt) and induced (+Dox) or un-induced (−Dox) *TgOct4* transgenic (TG) somitogenesis embryos analysed by whole mount *in situ* hybridisation. Middle: High magnification images depicting *Sox2* expression in the neural tube. *Arrow*: Ectopic lateral *Sox2* expression in induced embryo. Bottom: representative sections showing *Sox2* expression in the wild type (*left*) and the induced transgenic (*right*) embryos. The position of the sections relative to the embryo is indicated by the lines on the *top left* image. *Arrow*: Ectopic *Sox2* expression in induced embryo. **c**-**e** Log expression levels of *Oct4, Nanog* and *Sox2* in pooled pre- and somitogenesis stage *TgOct4* embryos in the presence (*white bars*) or absence of Doxycycline (*black bars*). Error bars represent s.e.m. (*n* = 3). Note error bars in (**c**) are smaller than the resolution of the figure. In all cases Oct4-induced embryos were treated with Doxycycline for 24 h prior to the indicated recovery time point. E14, E14tg2a ES cells; MEF, mouse embryonic fibroblasts; PS, prestreak; ES, early streak; OB, no allantoic bud; LB, late allantoic bud; LHF, late headfold; s, somite

Given the presence of several critical reprogramming factors in somatic cells of somitogenesis stage embryos we next tested the effects of restoring the expression of the principal pluripotency determinant *Oct4* during these stages. We employed a mouse line (*TgOct4*) carrying an *Oct4* transgene that is ubiquitously expressed in response to Doxycycline (Dox) administration [22]. We have previously shown that ectopic *Oct4* induction in this line at somitogenesis stages leads to reactivation of the pluripotency indicator *Nanog* within 24 h (hr) after explantation in EpiSC culture conditions and subsequent re-establishment of pluripotency evidenced by the derivation of stable EpiSC lines and formation of teratocarcinomas from grafts of tissue pieces to the adult kidney capsule [3]. We thus tested whether pluripotency is reactivated in Dox-treated embryos *in utero*. A 24 h treatment of pregnant females with Dox led, as expected, to the robust upregulation of *Oct4* in transgenic embryos (Fig. 3a, c), and wholemount *in situ* hybridisation showed widespread *Oct4* expression (Fig. 3a). However, we observed no reactivation of Nanog expression, as assessed either visually using a Nanog-GFP reporter transgene (data not shown), or by qPCR (Fig. 3d). A slight upregulation of *Sox2* in the lateral area of the somites was detected (Fig. 3b), but otherwise there was no ectopic *Sox2* expression (Fig. 3b, e). These results indicate that ubiquitous induction of ectopic *Oct4* expression in somitogenesis-stage embryos does not result in widespread reactivation of the pluripotency transcriptional network.

**Fig. 4:**
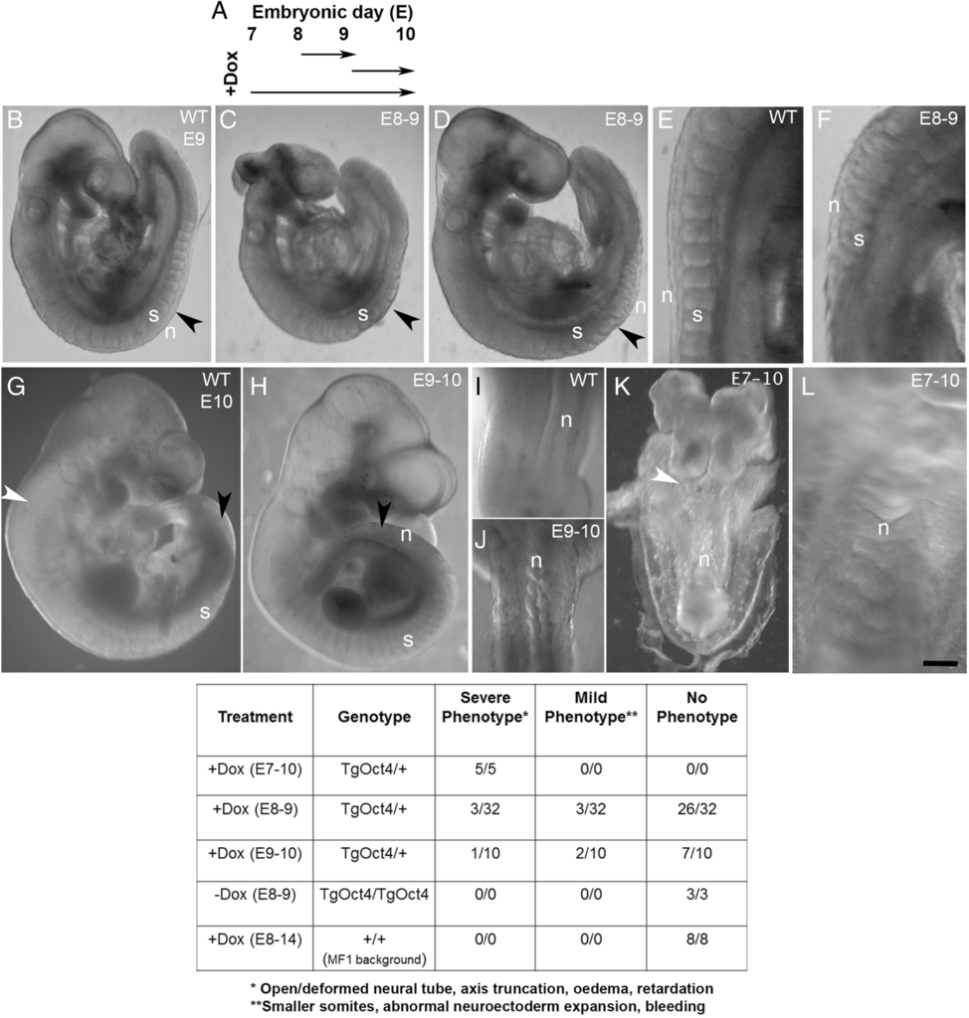
Phenotypic consequences of ubiquitous ectopic Oct4 expression. **a** Timings/duration of Dox administration. **b**-**f** Wildtype untreated (**b**, **e**) or 24 h Dox-treated TgOct4 embryos (**c**, **d**, **f**) recovered at E9.5. (**e**, **f**) high magnification views of wildtype (**e**) or Dox-treated TgOct4 embryos showing affected somites. **g**-**j** Wildtype untreated (**g**, **i**) or 24 h Dox-treated TgOct4 embryos (**h**, **j**) recovered at E10.5. (**i**, **j**) high magnification views of wildtype (**i**) or Dox-treated TgOct4 embryos showing affected neural tube. **k**, **l** 72-h Dox-treated TgOct4 E10.5 embryo. **l** high magnification view showing kinked neural tube. WT, wild type; s, somite; n, neural ectoderm. *Arrowheads* indicate the position of the somite pair formed at the time of Oct4 induction. *Black arrowheads*: 24 h induction (e8-9 or e9-10); *white arrowheads*: induction from e7-10. Scale bar: 500 μm (**b**-**d**); 250 μm (**e**, **f**, **i**-**l**); 800 μm (**g**, **h**). The observed phenotypes are summarised in the table

Although Dox-treated transgenic embryos showed no evidence of pluripotency network reactivation, they exhibited consistent developmental abnormalities (Fig. 4). The most prominent was a failure of dorsal closure of the anterior neural tube (Figs. 3b, 4). Similar brain malformations have been reported by [23] after ubiquitous Oct4 expression. However, we also observed a phenotype in the posterior parts of the axis not reported by these authors. Therefore we initiated a series of experiments to explore this phenotype. Treatment of heterozygous *TgOct4* embryos (after mating with wild type mice) from E7.5-E10.5 led to severe retardation with very little somitogenesis evident (Fig. 4). Shorter treatments from E8.5-E9.5 or E9.5-E10.5 produced phenotypes in the posterior part of the axis with little perturbation to the axial regions formed prior to treatment (Fig. 4). In axial regions formed after Dox administration, a kinked neural tube, suggestive of excess neural tissue, and small somites were evident (Figs. 3b, 4). Therefore, ectopic Oct4 expression during somitogenesis disrupts axis elongation, and in particular may interfere with the balance of neural and mesodermal tissues emanating from the TB region.

### Development of a novel T(Bra)-based tool for inducible gene expression in the PS and tail bud

**Fig. 5:**
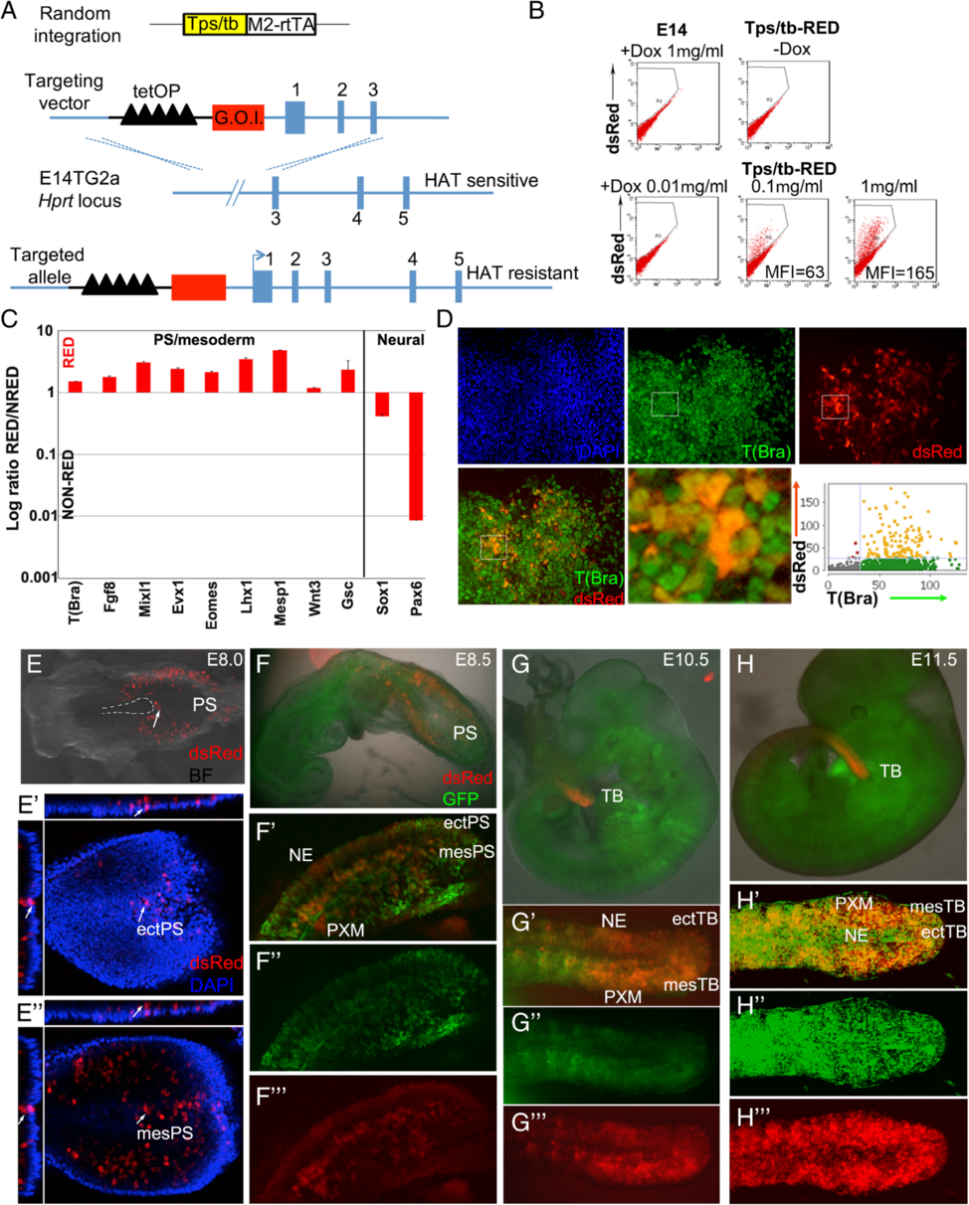
PS-specific induction of dsRed2. **a** Schematic diagram depicting the targeting strategy for the construction of the Tps/tb-RED ES cell line. Correctly targeted clones reconstruct a functional *Hprt* locus and these are therefore resistant to HAT selective medium. Numbers indicate Hprt exons. HAT, hypoxanthine-aminopterin-thymidine. G.O.I. gene of interest; P, promoter; TetOP, tet operator sequences. **b** Flow cytometry analysis of dsRed2 expression in Tps/tb-RED ES cells cultured in the absence of LIF and the presence of varying concentrations of Dox for four days. E14, E14tg2a; MFI, Mean Fluorescence Intensity. **c** qPCR expression analysis of indicated markers in sorted dsRed2^+^ and dsRed2^−^ differentiated Tps/tb-RED ES cells. Results are represented as log_10_ ratio of expression versus dsRed2^−^ cells. Error bars represent the S.E.M (*n* = 3). **d** Immunocytochemical analysis of T(Bra) expression in differentiated Tps/tb-RED ES cells cultured in the presence of Dox. Its correlation with dsRed2 expression as assessed by single cell image analysis is shown in the graph on the right. **e** dsRed2 expression in the PS of a Dox-treated chimeric E8.0 Tps/tb-RED embryo. The broken line area denotes the node and emergent notochord; the arrow indicates a labelled cell cluster in the node-streak border. (*e’-e”*) Representative optical confocal image sections of the posterior region of a chimeric E8.0 Tps/tb-RED embryo showing the presence of dsRed2^+^ cells in the ectoderm (*e’*) and mesoderm (*e”*) (*arrows*). Cell nuclei were stained with 4',6-diamidino-2-phenylindole (DAPI). (**f**-*f’”*) dsRed2 induction in the PS of Dox-treated E8.5 chimeric C2 (Tps/tb-RED/CAG-GFP) ES derived embryos. GFP expression marks chimeric contribution. NE, neuroectoderm; PXM, paraxial mesoderm. (**g**-**h**) dsRed2 expression in the tail bud of Dox-treated E10.5 (**g**-*g”’*) and E11.5 (**h**-*h”’*) C2 chimeric embryos. In all cases chimeric embryos were treated with Doxycycline for at least 24 h prior to the indicated recovery time point

The severity of the embryonic phenotype produced by ubiquitous Oct4 expression precluded assessment of tumour formation over a longer period. Therefore we developed a system that would allow targeted and inducible transgene expression specifically at the midline of the extending axis, i.e. the site where extragonadal teratomas are most often observed in humans (Fig. 1b). We established a feeder-free E14tg2a-derived ES cell line which contains a randomly integrated cassette (Tps/tb-rtTA) consisting of the PS/tb-specific 1.2 kb T(Bra) promoter fragment [24] driving the reverse tetracycline transactivator rtTA2^S^-M2 gene [25]. To conditionally express heterologous genes, we targeted a Tetracycline Responsive Element (TRE) module coupled to a gene of interest at the *Hprt* locus (Fig. 5a). This strategy has been shown to be very efficient for achieving introduction of single copy transgenes into a locus offering a defined chromatin environment for stable ectopic gene expression [26].

We tested the spatiotemporal specificity of our system by examining the Dox-dependent expression of a TRE-dsRed2 fluorescent reporter introduced into the *Hprt* locus in the Tps/tb-rtTA ES cell line. We first investigated the dynamics of dsRed2 induction in vitro by culturing the resulting Tps/tb-rtTA/HPRT^TRE-dsRed2^ reporter ES cell line (hereafter referred to as Tps/tb-RED) in the absence of LIF and in presence of serum. We found that these conditions promote the induction of T(Bra) positive, PS-like cells within 3–4 days (Additional file 1: Figure S1) in line with previously published data [27–29]. Culture of Tps/tb-RED ES cells in –LIF/+serum media and in the presence of Dox promoted the induction of dsRed2^+^ cells with dsRed2 expression peaking at day 4 of differentiation (17.5 ± 2.7 %; Fig. 5b). A 10-fold reduction in the levels of Dox correlated with a decreased induction of dsRed2^+^ cells (5.75 ± 1.8 %), Mean fluorescence intensity also decreased at low Dox concentrations (63 at 0.1 μg/ml compared to 165 at 1 μg/ml) indicating that our inducible gene expression system exhibits a degree of tunability (Fig. 5b). We observed no leakiness in dsRed2 expression in the absence of Dox (Fig. 5b).

To determine the identity of dsRed2-expressing cells on day 4 after LIF removal in vitro, the expression of PS markers was examined in flow sorted dsRed2^+^ and dsRed2^−^ populations. Sorted dsRed2^+^ cells were enriched for all PS markers tested compared to their dsRed2^−^ counterparts (Fig. 5c). Conversely, expression of neural markers such as Sox1 and Pax6 was underrepresented in dsRed2^+^ cells (Fig. 5c). All dsRed2^+^ cells were also T(Bra)-immunopositive, although T(bra) expression was more widespread than that of dsRed2 (Fig. 5d). This suggests that at least some T(Bra)^+^/dsRed2^−^ cells may have a ventral node/notochord identity, since the promoter is inactive in this population. However, we cannot exclude the possibility that these cells may also reflect a lower sensitivity of detection of dsRed fluorescence or differences in turnover between the dsRed2 and T(Bra) proteins. Collectively, these results indicate that dsRed2 induction takes place in vitro specifically within cells exhibiting a PS/mesodermal identity and are consistent with our previous findings that the Tps/tb promoter becomes activated within the PS quite early, at the beginning of gastrulation [30].

We investigated the induction of the dsRed2 reporter in Dox-treated somitogenesis-stage (E8.5-11.5) chimeric embryos generated using Tps/tb-RED ES cells modified to carry a ubiquitously-expressed GFP transgene (cell line C2). We found that dsRed2 was expressed in the PS, tail bud and recently emerged mesoderm at all stages tested (Fig. 5e-h). At E8.5, expression was present up to and including the epiblast close to the node (in the node-streak border), but absent in the ventral node and notochord (Fig. 5e). While early on E8.5 (<5 somites), expression was almost exclusively mesodermal, later E8.5 embryos (>8 somites) showed expression in emerging neurectoderm as well as mesoderm (Fig. 5e-f). At E10.5-11.5, dsRed2 fluorescence was visible in both the ectodermal and mesodermal components of the TB as well as the emerging neurectoderm and mesoderm (Fig. 5g-h). The highest intensity dsRed2 expression was seen in the presomitic mesoderm, consistent with the relatively long maturation time of this fluorescent protein [31]. Thus the expression of dsRed2 accurately reports activation of T(Bra) expression in the PS and TB and is in agreement with previously published findings [28, 32, 33]. Examination of the anterior limits of expression at E8.5 and E10.5-11.5 suggests that dsRed2 protein persists for around 24 h after its initial transcriptional activation in the PS and TB. Together these data indicate that our transgenic system comprises a reliable platform for inducible heterologous gene expression in the PS and TB.

### Expression of Oct4 and Nanog in the primitive streak/tail bud after the start of somitogenesis does not result in neoplasia

**Fig. 6:**
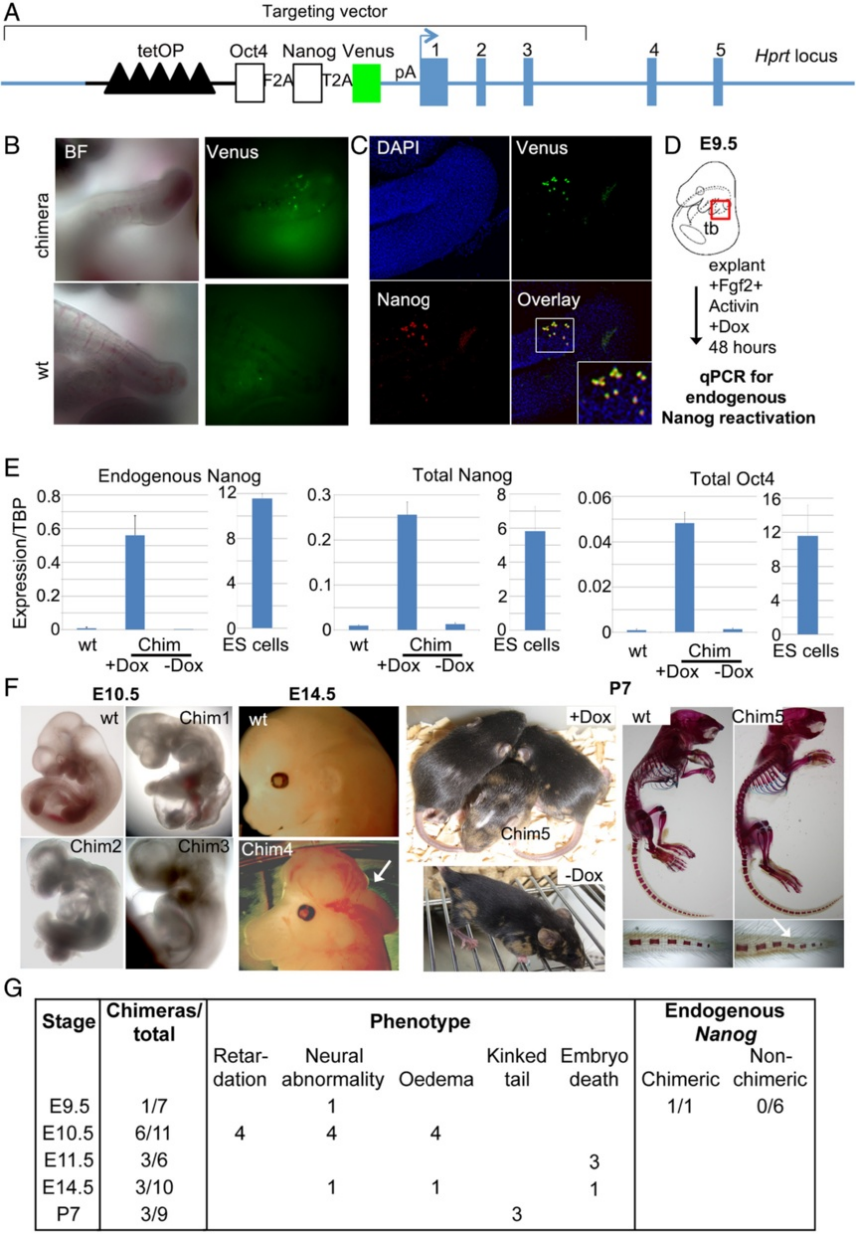
Ectopic induction of pluripotency factors in the PS/tail bud. **a** Diagram showing the components of the Dox-inducible pluripotency factor expression cassette targeted to the Hprt locus. Numbers indicate Hprt exons. TetOP, tet operator sequences; P, promoter. **b** Venus expression in the tail bud of an E10.5 wt a Tps/tb-Oct-Nanog-Venus chimeric embryo treated with Dox at E8.5. **c** Nanog and Venus expression in the tail bud of an E10.5 wt a Tps/tb-Oct-Nanog-Venus chimeric embryo treated with Dox at E8.5 following immunostaining with a anti-Nanog antibody. **d** Experimental strategy employed for the derivation of tail bud EpiSC explants from Tps/tb-Oct-Nanog-Venus chimeric embryos. **e** qPCR expression analysis of Oct4, Nanog and endogenous Nanog expression in Dox-treated, untreated and wild type (wt) EpiSC explants as well as ES cells. For detection of endogenous Nanog expression a set of primers complementary to the Nanog 5’UTR region were employed as previously described [34]. Expression is shown as relative to TBP. Error bars represent s.d. **f** Morphology of Tps/tb-Oct-Nanog-Venus chimeric embryos and P7 pups after continuous Dox treatment starting at E8.5. A wild type embryo and an untreated chimeric pup are also shown as controls. The arrowhead in the E14.5 embryo image indicates exencephaly. Far right: skeletal preps of a wild type and a chimeric P7 pup. The arrowhead indicates a kink in the tail of the chimeric embryo. **g** Summary of the phenotypes observed in Tps/tb-Oct-Nanog-Venus chimeric embryos after continuous Dox treatment starting at E8.5. These correspond to each of the chimeric embryos recorded in the second column of the table with the exception of the E10.5 chimeras where the same 4/6 chimeric embryos exhibited the phenotypes noted in the panel and the remaining two chimeric embryos appeared normal. The E9.5 chimeric embryo exhibited endogenous Nanog reactivation following explantation in EpiSC conditions. In all cases Doxycycline administration started at E8.5

We next examined whether expression of pluripotency factors in the primitive streak /tail bud after gastrulation is sufficient to reactivate pluripotency *in vivo* potentially leading to the formation of teratocarcinomas in the midline. We introduced a TRE-Oct4-2A-Nanog-2A-Venus cassette into the *Hprt* locus in Tps/tb-rtTA ES cells (Fig. 6a) thus allowing the simultaneous expression of three proteins: Oct4, Nanog and Venus, within cells in which the Tps/tb promoter is active.

Differentiation of Tps/tb-rtTA-Oct-Nanog-Venus ES cells in the absence of LIF resulted in robust induction of Venus expression correlating with the emergence of PS-like cells in vitro (Additional file 2: Figure S2). These ES cells were injected into blastocysts and chimeras were recovered during gestation or after development to term. Dox treatment starting at E8.5 drove efficient Venus expression and transgene induction in the PS/TB (Fig. 6b, c). Since we previously showed that Nanog reactivation serves as an early indicator of successful pluripotent EpiSC derivation [3], we tested whether induction of these transgenes in explants reactivated endogenous Nanog on culture in Activin/Fgf EpiSC growth conditions. Explantation of E9.5 chimeric tail buds to EpiSC culture in the presence of Dox led to reactivation of endogenous Nanog, revealed by qPCR using primers against the 5’UTR of the Nanog locus [34] which is absent from the Tps/tb-Oct4-Nanog-Venus vector, (Figure 6e), showing that Oct4 was active in this system. Most Dox-treated chimeric embryos exhibited developmental defects reminiscent of those observed after ubiquitous Oct4 induction at midgestation: open neural tube, oedema, and kinked posterior neural tube (Fig. 6f, g). Some Dox-treated chimeras were born apparently healthy although skeletal preparations revealed small kinks in the tail (Fig. 6f) while one animal exhibited hindlimb polydactyly (data not shown). Since we also observed the same polydactylous phenotype in one untreated control chimera (data not shown) this phenotype is most likely to be unrelated to the induction of the Oct4/Nanog/Venus transgenes. Importantly, we did not observe any teratomas in Dox-treated liveborn chimeric animals (Fig. 6f).

In conclusion, we find no evidence that ectopic expression of Oct4 and Nanog within the PS and TB during mid-late gestation results in tumour formation *in vivo*. Although both ubiquitous Oct4 expression, and tail bud-specific Oct4/Nanog expression reactivated pluripotency in vitro in EpiSC culture, their embryonic expression neither reactivated endogenous Nanog nor led to or the development of teratomas.

## Discussion

### Embryonic environment and tumorigenicity of pluripotent cells

Human teratomas/teratocarcinomas typically occur in the gonads of postpubertal individuals and hence are classed as germ cell tumours and considered of germ cell origin [8]. However in prenatal and neonatal infants, teratomas most frequently occur in extra-gonadal locations [35, 36]. Since around 50 % of sacrococcygeal teratomas are detectable by ultrasound scan at 18–20 weeks [36], these tumours may arise early during gestation. The predominant view regarding their aetiology is that PGCs fail to migrate properly towards the genital ridges and through evasion of apoptosis give rise to germ cell tumours [7, 8, 37]. However, the occurrence of extra-gonadal teratomas in locations distant from the canonical route of germ cell migration (i.e. the brain and mediastinum) raises the possibility that at least some may be of a somatic cell origin. Since pluripotent cells of somatic origin are capable of normal development in gastrulation-stage embryos [15], yet form teratocarcinomas in an adult environment [38, 39], there must be a stage at which pluripotent cells become neoplastic. Here we provide proof-of-principle that introduction of established pluripotent cells into midgestation (E9.5) embryos after normal pluripotency shutdown leads to the development of teratomas. Thus, the midgestation environment is prohibitive for the integration of established pluripotent cells, and permissive for their growth as teratocarcinomas.

Embryonic pluripotency is extinguished *in vivo* around the start of somitogenesis, coincident with a rapid downregulation first of Nanog and then of Oct4 expression [3]. Oct4 is critical for the maintenance of a pluripotent state in the postimplantation epiblast and ectopic Oct4 reactivation in post-pluripotent primary embryonic tissue after grafting beneath the adult kidney capsule leads to teratocarcinoma formation [3]. This led us to hypothesise that the failure of embryonic somatic cells to shut down Oct4 might result in extragonadal teratomas. However, we did not observe tumour formation following ectopic induction of Oct4 alone or in combination with Nanog even at the posterior of the embryo, a site where the other main reprogramming factors, Sox2, Klf4 and c-Myc were found to be present (Fig. 2 and [21]). So far only cells that express pluripotency gene regulatory network factors, including Oct4, Sox2 and Nanog, have been shown to produce teratocarcinomas. In adult mice, expression of Oct4 alone leads to atypical epithelial neoplasias [22], whereas expression of all four reprogramming factors produces teratocarcinomas [12, 13]. Thus it is likely that a cell-of-origin of spontaneously arising teratomas would express all these genes or their functional equivalents. Both ubiquitous Oct4 [3] and ps/tb-specific Oct4/Nanog (Fig. 6e) are sufficient to promote the reassembly of the pluripotency network in EpiSC culture, as evidenced by the reactivation of endogenous Nanog expression [3]. However, neither of these perturbations resulted in the *in vivo* reactivation of the pluripotency network or teratoma formation *in vivo*. This reinforces the conclusion that a permissive environment, as well as the expression of intrinsic factors, is important for the formation of teratomas.

The extracellular environment is known to be important in determining the behaviour of pluripotent cells. Mouse ES cells readily incorporate into preimplantation embryos at the stage from which they were isolated [14] and contribution to the resulting chimeras suggests that they respond correctly to differentiation cues provided by the embryo. Similarly, EpiSCs efficiently colonise cultured gastrulation-stage embryos [15]. However, ES cells cannot integrate in post-implantation embryos [15] while EpiSCs are incompatible with either early somite-stage embryos from which pluripotency is lost [15] or pre-implantation embryos [39]. Moreover, either pluripotent cell type, when introduced into adults, forms teratocarcinomas at very high frequencies. Thus, the ability of the organism to suppress neoplasia in a pluripotent cell type depends critically on the extracellular environment.

The failure of pluripotent cells to integrate correctly into a normal environment may be a necessary early step in the formation of a niche that is permissive for pluripotent cell maintenance. Such a niche could either be supplied by the surrounding embryonic cells, or by the pluripotent cells themselves, for example via the ability of differentiated derivatives of pluripotent cells to secrete self-renewal factors [40]. The latter possibility provides a potential explanation for the ability of established lines to form tumours in midgestation embryos, while expressing Oct4 *in situ* at the same stage does not. Together with the observation that fresh tissue grafted to the adult kidney capsule leads to teratocarcinoma formation, this suggests that the E8.5-9.5 embryonic environment lacks factors that promote a pluripotency-permissive niche.

What signalling pathways could constitute a pluripotency-permissive niche? In vitro, Oct4-induced Nanog reactivation in somitogenesis-stage EpiSC explants and subsequent acquisition of pluripotency is dependent on Nodal/activin signalling [3]. At the beginning of gastrulation, Nodal signalling is widespread in the posterior region of the embryo, but becomes highly restricted to the node coincident with the extinction of embryonic pluripotency at early-somite stages, and is absent after 7–8 somites [41]. Similarly, activin subunit transcripts are absent from the embryo proper during the critical period of pluripotency shutdown, till around E9.5, after which restricted expression domains appear in various regions [42, 43]. Moreover, Nodal signalling has been shown to be critical for regulating germ cell pluripotency [44] and perturbations in its levels have been linked to the formation of germ cell tumours, including extragonadal ones [44–47].

### Ectopic Oct4 expression perturbs normal development

The phenotypes produced by Oct4 expression are reproducible and dependent on the stage at which expression was induced. Ubiquitous Oct4 overexpression before closure of the cranial neural folds leads to an open brain phenotype, while expression thereafter affects mainly the parts of the anteroposterior axis that form after induction of the Oct4 transgene, where somites are smaller than normal and the neural tube is kinked. Induction of Oct4/Nanog in the PS/TB leads to rather similar phenotypes (failure of cranial neural tube closure and kinked posterior regions of the axis), suggesting both that the phenotypes observed are mainly due to Oct4 expression, rather than Nanog, and that these phenotypes result from ectopic pluripotency factor expression in the PS/TB. The combination of small somites and a kinked neural tube suggests that Oct4 expression during somitogenesis may hinder the production or expansion of mesoderm at the expense of neurectoderm. Interestingly, depletion of Oct4 around E7.5 severely affects both neural tube closure and somitogenesis/posterior development and results in misregulation of a number of key signalling pathways such as TGFβ and Notch [48]. Together, these findings suggest that Oct4 plays a critical role in morphogenetic processes possibly by influencing proliferation [48] and/or adhesion [49].

### A transgenic tool for inducible expression in the PS and the tail bud

Various transgenic strategies for T-dependent expression have been reported, including targeted replacements of the T gene [27], cells and mice containing integrated copies of cosmid [32, 50] or BAC constructs [29] spanning the T genomic region, and up to 2.4 kb upstream of the transcription start site [28, 32, 51]. The expression of our reporter in the primitive streak but not node and notochord is consistent with these studies that show up to 8.3 kb of upstream sequences contain only these regulatory elements. The expression of the dsRed2 and Oct/Nanog transgenes specifically in the primitive streak and tail bud and their nascent mesoderm derivatives demonstrates the utility of T-rtTA driver for PS and tail bud-specific expression of heterologous genes in vitro and *in vivo*. Targeting heterologous genes at the *Hprt* locus offers a convenient selectable strategy allowing integration of a single transgene copy at a single site in a permissive chromosomal domain thus eliminating unpredictable chromosomal position effects on expression due to random integration.

## Conclusions

We have shown that established pluripotent cells are neoplastic if present after the time when pluripotency is normally shut down, i.e. at organogenesis stages. We also show that reactivation of Oct4, or Oct4 and Nanog, after pluripotency shutdown is not sufficient to reactivate pluripotency *in vivo*, and requires additional signals from the cellular microenvironment. It will be interesting in future to determine whether sites at which either Activin/Nodal/Fgf or LIF/BMP signalling is naturally present coincide with sites at which teratomas spontaneously arise in mice and humans.

## Methods

### Generation of reporter ES cell lines

To construct the Tps/tb-RED ES cell line, we first engineered a transgene in which a 1.2 kb Brachyury (T) proximal upstream regulatory element and promoter fragment [24] was linked to the sequence of the reverse tetracycline transactivator rtTA2^S^-M2 [25]. This transgene, Tps/tb-rtTA, was randomly integrated by electroporation into E14Tg2a (129 background) ES cells. The resulting clones were screened for optimal expression levels of rtTA after LIF removal. The tet-responsive element (TRE) upstream of the dsRed2 fluorescent reporter gene or the Oct4-F2A-Nanog-T2A-Venus cassette were then introduced, by homologous recombination at the *Hprt* locus, into the clone expressing the highest levels of T-rtTA mRNA. Correctly targeted clones reconstruct a functional *Hprt* locus and were identified by selection in medium with hypoxanthine, aminopterin and thymidine (HAT) to generate Tps/tb;Hprt^TRE-dsRed2^ or Tps/tb;Hprt^TRE-Oct/Nanog/Venus^. The HAT resistant clones with the most robust induction of dsRed2 or Venus were then employed for detailed analysis. Subclones of the Tps/tb-RED ES cell line were also generated by introducing a randomly-integrated CAG-GFP plasmid and identifying clones with ubiquitous expression. A representative clone of each resulting ES cell line (Tps/tb;Hprt^TRE-dsRed2^, Tps/tb;Hprt^TRE-Oct/Nanog/Venus^ or Tps/tb;Hprt^TRE-dsRed2^-CAG-GFP, (named C2)) was used to generate chimeric embryos.

### Cell culture and differentiation

Feeder-free ES cells were routinely cultured in gelatinised flasks in GMEM-based medium supplemented with LIF. For induction of PS-like cells in vitro, ES cells were seeded at a density of 7.5×10^3^ cells/cm^2^, cultured in ES medium without LIF for up to four days in the presence of Doxycycline (0.01-1 μg/ml; Sigma).

### Mouse husbandry and *in vivo* transgene induction

Chimeric embryos were generated either by morula aggregation or blastocyst injection followed by transfer into pseudopregnant foster (CBA/BL6 F1) mothers using standard procedures. For transgene induction oral doxycycline was administrated at a final concentration of 1–2 mg/ml in drinking water containing 5 % sucrose. Mice were maintained on a 12 h light/12 h dark cycle. For timed matings, noon on the day of finding a vaginal plug was designated E0.5. To assess the effects of ectopic Oct4 activation, transgenic TgOct4 mice were either treated directly with Doxycyline or first crossed with wild type (129 or MF1 background) or Nanog:GFP [129-Nanog^tm1(GFP-IRES-Puro)^] mice [52] before Doxycycline administration to pregnant mothers. ES cell suspensions were manually injected into the amniotic cavity of E9.5 embryos *in utero* using a hand-pulled Pasteur pipette after laparotomy and exposure of the uterine horns under a general anaesthetic. The position of the amniotic cavity was visualised by trans-illumination with a fibre-optic light.

### Microdissection of tail bud regions

Tail bud regions were microdissected with hand-pulled solid glass needles as described previously [20] and 7–10 regions pooled for RNA isolation.

### *In situ* hybridization

Whole-mount *in situ* hybridisation was performed as described [53]; proteinase K treatment was empirically adjusted between 8–16 min according to embryo size and stage. The riboprobes used were: Oct4 [54], Sox2 [55] and Klf4 which was designed by cloning a 900 bp fragment (base pair 972–1871, NM_010637.3) into a pCR®II-TOPO® vector (Life technologies). Embryos were sectioned in paraffin wax (7 μm).

### Flow cytometry

Analysis of dsRed2 or Venus expression was performed using a FACS Calibur (BD Biosciences) cytometer. Cell sorting was performed using a FACSAria (BD Biosciences).

### EpiSC explantation

EpiSC explantation was performed as previously described [3]. Briefly, after removal of extraembryonic membranes, the tail buds of chimeric embryos were dissected, dissociated in trypsin/pancreatin and explanted in EpiSC medium [39] supplemented with 20 ng/ml activin A (PeproTech) and 10 ng/ml bFGF (R&D) in the presence (1–3 μg/ml) or absence of Doxycyline on irradiated mouse embryonic fibroblasts (MEFs). The duration of culture under these conditions was 48 h.

### Gene expression analysis

RNA isolation was performed using the RNeasy Mini or Micro kit (Qiagen) following the manufacturer’s instructions. On-column DNase I (Qiagen) digestion was carried out in all cases to eliminate genomic DNA contamination. The concentration and the purity of each sample were determined by UV spectroscopy using a NanoDrop ND-1000 (Thermo Scientific). Total RNA (25–250 ng) was reverse-transcribed into cDNA using random primers (Promega) and the SuperScript III reverse transcriptase kit (Invitrogen). Real-time RT-PCR was performed using a LightCycler 480 (Roche) and LightCycler 480 SYBR Green 1 Master Mix (Roche). All primers employed were designed to flank introns or span exon-intron boundaries and their sequences can be found in Additional file 3: Table S1. The optimal performance of each primer set was confirmed using 10-fold serial dilutions of plasmid preparations containing the corresponding target sequences. Serial dilutions of the targets were also employed to generate standard curves for determining copy numbers and values were normalized against the input determined for the housekeeping gene TATA-binding protein (TBP). All qPCR experiments were carried out using three technical replicates and at least two biological replicates.

### Immunostaining

Immunostaining of cells and embyros was performed as described previously [3]. The following primary antibodies were used: anti-Brachyury (N-19, Santa Cruz, 0.4 μg/ml), anti-Foxa2 (M-20, Santa Cruz, 2 μg/ml), anti-Gsc (N-12; Santa Cruz, 2 μg/ml), anti-Nanog, 2.5 μg/ml (14-5761-80, eBioscience).

### Imaging

Images were captured using a Zeiss Stemi SV11 or Nikon AZ100 dissecting microscope (for freshly dissected whole and *in situ* hybridised embryos), an Olympus IX51 (for cultured cells), or an Olympus BX61 (for sections). Image processing was performed using Adobe Photoshop software. For higher resolution visualisation of dsRed2 or Venus/Nanog expression a Leica TCS SP2 inverted confocal microscope was employed (Leica Microsystems). Image acquisition and processing were carried out using the Leica Confocal (Leica Microsystems) and Volocity (Improvision) software packages, respectively. Nuclear segmentation and fluorescence signal quantification were performed as described previously [3].

### Skeletal preparations and histology

Skeleton preparations and histological stains of tumour sections were prepared as described in [56].

## Ethics approval

All animal experiments were carried out in accordance with the UK Animals (Scientific Procedures) act 1986 under Home Office Licence 60/4435. Experiments were approved by the University of Edinburgh Animal Welfare Ethical Review Board.

## Additional files

Additional file 1: Figure S1. ES cells differentiated in the absence of LIF give rise to PS-like cells in vitro. (A) Time-course qPCR expression analysis of ES- and PS/organiser-specific markers in wild type E14 ES cells cultured in the presence of serum and absence of LIF for the indicated amount of time. (B) Immunocytochemistry of PS/organiser marker expression in wild type E14 ES cells cultured in the presence of serum and absence of LIF for 4 days. (JPEG 943 kb) Additional file 2: Figure S2. The induction of PS-like cells in vitro correlates with Venus upregulation in Tps/tb-Oct-Nanog-Venus ES cells. (A) Flow cytometry analysis of Venus expression in Tps/tb-Oct-Nanog-Venus ES cells after 4 days of differentiation in the presence of serum/Dox and absence of LIF. (B) Fluorescence microscopy of Venus expression Dox-treated and untreated Tps/tb-Oct-Nanog-Venus ES cells after 4 days of differentiation in the presence of serum/Dox and absence of LIF. (JPEG 607 kb) Additional file 3: Table S1. Primer sequences. (DOCX 14 kb)

## Abbreviations

ES: Embryonic stem
EpiSC: Epiblast stem cell
EC: Embryonal carcinoma
PGCs: Primordial germ cells
TB: Tail bud
CNH: Chordoneural hinge
NM: Neuromesodermal
Dox: Doxycycline
TRE: Tetracycline responsive element
PS: Primitive streak
