# Supplementary figures and images for: Intrinsic factors and the embryonic environment influence the formation of extragonadal teratomas during gestation
Source: BMC Dev Biol. 2015 Oct 9;15:35. doi: 10.1186/s12861-015-0084-7 (PMC4599726; doi:10.1186/s12861-015-0084-7)

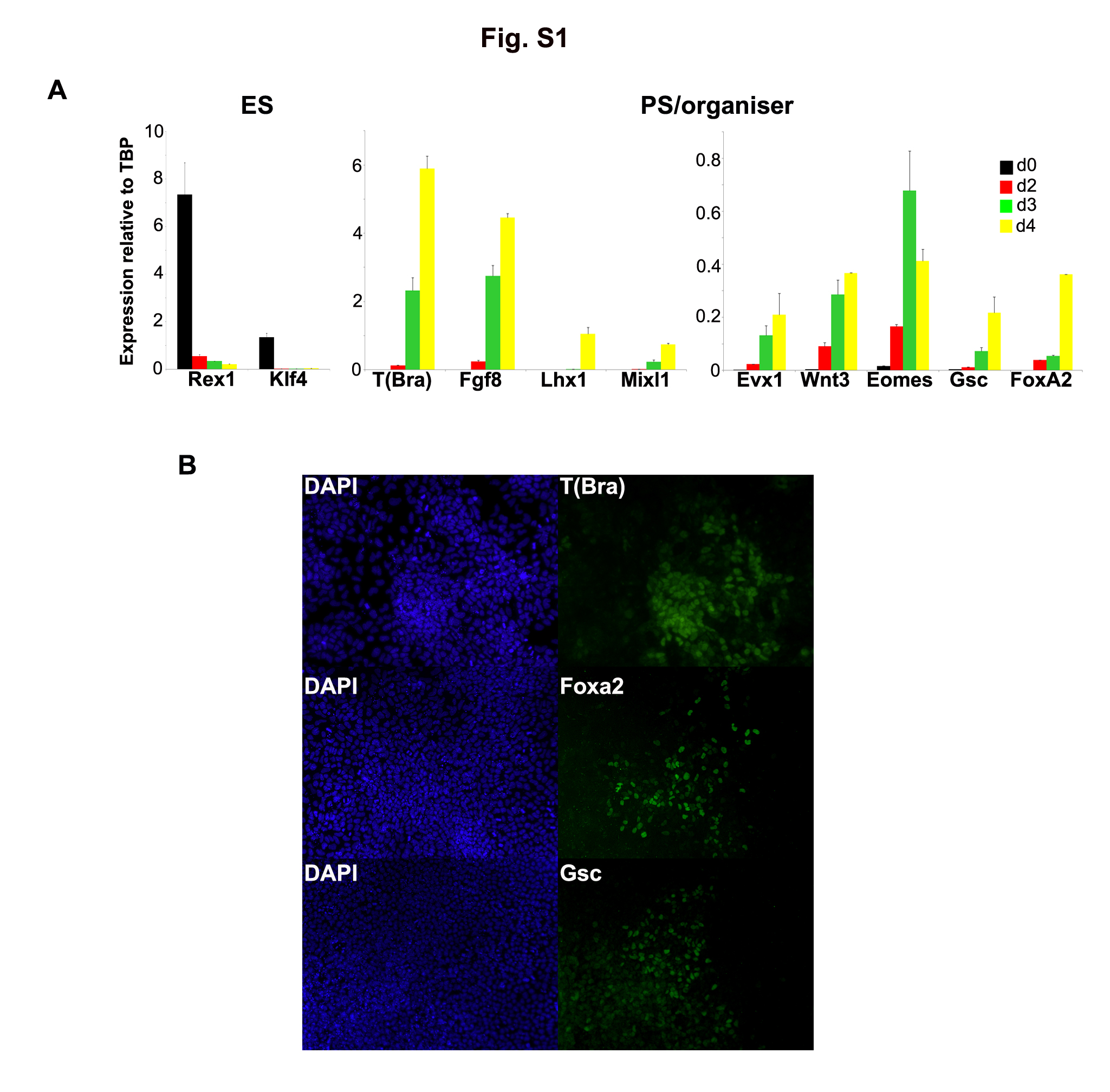

Supplement: Additional file 1: Figure S1. — ES cells differentiated in the absence of LIF give rise to PS-like cells in vitro. (A) Time-course qPCR expression analysis of ES- and PS/organiser-specific markers in wild type E14 ES cells cultured in the presence of serum and absence of LIF for the indicated amount of time. (B) Immunocytochemistry of PS/organiser marker expression in wild type E14 ES cells cultured in the presence of serum and absence of LIF for 4 days. (JPEG 943 kb) [file 12861_2015_84_MOESM1_ESM.jpg]

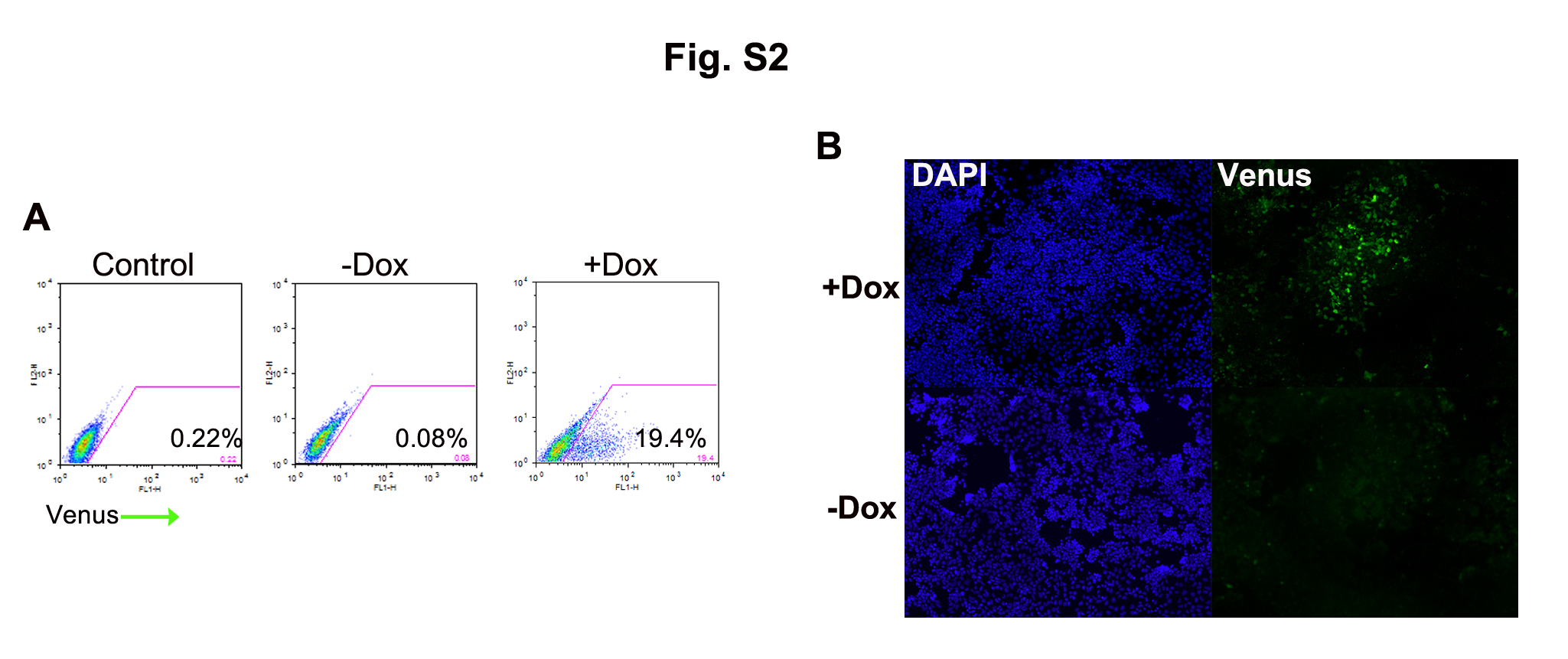

Supplement: Additional file 2: Figure S2. — The induction of PS-like cells in vitro correlates with Venus upregulation in Tps/tb-Oct-Nanog-Venus ES cells. (A) Flow cytometry analysis of Venus expression in Tps/tb-Oct-Nanog-Venus ES cells after 4 days of differentiation in the presence of serum/Dox and absence of LIF. (B) Fluorescence microscopy of Venus expression Dox-treated and untreated Tps/tb-Oct-Nanog-Venus ES cells after 4 days of differentiation in the presence of serum/Dox and absence of LIF. (JPEG 607 kb) [file 12861_2015_84_MOESM2_ESM.jpg]
