# Supplementary material for: Intrinsic factors and the embryonic environment influence the formation of extragonadal teratomas during gestation
Source: BMC Dev Biol. 2015 Oct 9;15:35. doi: 10.1186/s12861-015-0084-7 (PMC4599726; doi:10.1186/s12861-015-0084-7)
Supplement: Additional file 3: Table S1. — Primer sequences. (DOCX 14 kb) [file 12861_2015_84_MOESM3_ESM.docx]

| **Target gene** | **Forward Primer** | **Reverse Primer** |
| --- | --- | --- |
| Oct4 | GGCGTTCTCTTTGGAAAGGTGTTC | CTCGAACCACATCCTTCTCT |
| Nanog | CCTCCAGCAGATGCAAGAA | GCTTGCACTTCATCCTTTGG |
| Sox2 | GGCGGCAACCAGAAGAACAG | GCTTGGCCTCGTCGATGAAC |
| Klf4 | GGCGAGAAACCTTACCACTGT | TACTGAACTCTCTCTCCTGGCA |
| T(Bra) | GTGACTGCCTACCAGAATGA | ATTGTCCGCATAGGTTGGAG |
| Fgf8 | ATGGCAGAAGACGGAGACC | TTGTTCATGCAGATGTAGAGACC |
| Mixl1 | AGTTGCTGGAGCTCGTCTTC | AGGGCAATGGAGGAAAACTC |
| Evx1 | GTTTCAAGACCGCGGAGAT | TGACGCTTGTCCTTCATGC |
| Eomes | ACCGGCACCAAACTGAGA | AAGCTCAAGAAAGGAAACATGC |
| Lhx1 | CAGTGTCGCCAAAGAGAACA | TCAACGTCTCCAGTTGCTTG |
| Mesp1 | ACCCATCGTTCCAGTACGC | AGCATGTCGCTGCTGAAGA |
| Wnt3 | CGCTCAGCTATGAACAAGCA | GGTGTTTCTCCACCACCATC |
| Gsc | GAGACGAAGTACCCAGACGTG | GGCGGTTCTTAAACCAGACC |
| Sox1 | GTGACATCTGCCCCCATC | GAGGCCAGTCTGGTGTCAG |
| Pax6 | CGGAGGGAGTAAGCCAAGAG | TCTGTCTCGGATTTCCCAAG |
| Rex1 | ACGAGTGGCAGTTTCTTCTTGGGA | TATGACTCACTTCCAGGGGGCACT |
| Foxa2 | CATCCGACTGGAGCAGCTA | GCGCCCACATAGGATGAC |
| Nanog (5’UTR) | TGGCCTTCAGATAGGCTGAT | CAAGAAGTCAGAAGGAAGTGAGC |
| TBP | GGGGAGCTGTGATGTGAAGT | CCAGGAAATAATTCTGGCTCA |
